# A Gammaherpesvirus Noncoding RNA Is Essential for Hematogenous Dissemination and Establishment of Peripheral Latency

**DOI:** 10.1128/mSphere.00105-15

**Published:** 2016-03-02

**Authors:** Emily R. Feldman, Mehmet Kara, Lauren M. Oko, Katrina R. Grau, Brian J. Krueger, Junjie Zhang, Pinghui Feng, Linda F. van Dyk, Rolf Renne, Scott A. Tibbetts

**Affiliations:** a Department of Molecular Genetics and Microbiology and UF Health Cancer Center, College of Medicine, University of Florida, Gainesville, Florida, USA; b Department of Microbiology and Immunology, University of Colorado School of Medicine, Aurora, Colorado, USA; c Department of Molecular Microbiology and Immunology, Keck School of Medicine, University of Southern California, Los Angeles, California, USA; Boston University School of Medicine

**Keywords:** TMER, dissemination, herpesviruses, latency, miRNA, noncoding RNA, viral pathogenesis

## Abstract

Recent intense investigations have uncovered important functions for a diverse array of novel noncoding RNA (ncRNA) species, including microRNAs (miRNAs) and long noncoding RNAs. Not surprisingly, viruses from multiple families have evolved to encode their own regulatory RNAs; however, the specific *in vivo* functions of these ncRNAs are largely unknown. The human gammaherpesviruses Epstein-Barr virus (EBV) and Kaposi’s sarcoma-associated herpesvirus (KSHV) are highly ubiquitous pathogens that are associated with the development of a wide range of malignancies, including Burkitt’s lymphoma, Hodgkin’s lymphoma, nasopharyngeal carcinoma, and Kaposi’s sarcoma. Like EBV and KSHV, murine gammaherpesvirus 68 (MHV68) establishes lifelong latency in B cells and is associated with lymphoproliferative disease and lymphoma. Similar to the EBV-encoded small RNA (EBER)-1 and -2, MHV68 encodes eight 200- to 250-nucleotide polymerase III-transcribed ncRNAs called TMERs (tRNA-miRNA-encoded RNAs), which are highly expressed in latently infected cells and lymphoproliferative disease. To define the *in vivo* contribution of TMERs to MHV68 biology, we generated a panel of individual TMER mutant viruses. Through comprehensive *in vivo* analyses, we identified TMER4 as a key mediator of virus dissemination. The TMER4 mutant virus replicated normally in lungs and spread with normal kinetics and distribution to lung-draining lymph nodes, but it was significantly attenuated for infection of circulating blood cells and for latency establishment at peripheral sites. Notably, TMER4 stem-loops but not miRNAs were essential for wild-type TMER4 activity. Thus, these findings revealed a crucial miRNA-independent function of the TMER4 ncRNA in MHV68 hematogenous dissemination and latency establishment.

**IMPORTANCE** Noncoding RNAs (ncRNAs) represent an intriguing and diverse class of molecules that are now recognized for their participation in a wide array of cellular processes. Viruses from multiple families have evolved to encode their own such regulatory RNAs; however, the specific *in vivo* functions of these ncRNAs are largely unknown. Epstein-Barr virus (EBV) and Kaposi’s sarcoma-associated herpesvirus (KSHV) are ubiquitous human pathogens that are associated with the development of numerous malignancies. Like EBV and KSHV, murine gammaherpesvirus 68 (MHV68) establishes lifelong latency in B cells and is associated with lymphomagenesis. The work described here reveals that the MHV68 ncRNA TMER4 acts at a critical bottleneck in local lymph nodes to facilitate hematogenous dissemination of the virus and establishment of latency at peripheral sites.

**Podcast**: A podcast concerning this article is available.

## Abstract (precis)

Noncoding RNAs (ncRNAs) represent an intriguing and diverse class of molecules that are now recognized for their participation in a wide array of cellular processes. Viruses from multiple families have evolved to encode their own such regulatory RNAs; however, the specific *in vivo* functions of these ncRNAs are largely unknown. Epstein-Barr virus (EBV) and Kaposi’s sarcoma-associated herpesvirus (KSHV) are ubiquitous human pathogens that are associated with the development of numerous malignancies. Like EBV and KSHV, murine gammaherpesvirus 68 (MHV68) establishes lifelong latency in B cells and is associated with lymphomagenesis. The work described here reveals that the MHV68 ncRNA TMER4 acts at a critical bottleneck in local lymph nodes to facilitate hematogenous dissemination of the virus and establishment of latency at peripheral sites.

## INTRODUCTION

Murine gammaherpesvirus 68 (MHV68; also known as γHV68 or MuHV-4) is a rodent gammaherpesvirus that is genetically and pathogenically related to the ubiquitous and important human pathogens Epstein-Barr virus (EBV) and Kaposi’s sarcoma-associated virus (KSHV, also known as human herpesvirus 8 [HHV-8]). These gammaherpesviruses establish lifelong latent infections in B cells and are highly associated with the development of multiple types of malignancies, including Burkitt’s B cell lymphoma, Hodgkin’s lymphoma, nasopharyngeal carcinoma, and Kaposi’s sarcoma.

Noncoding RNAs (ncRNAs) have gained significant attention over the past decade due to the widespread use of next-generation sequencing to study their expression and rapid advances in decoding their functions in a multitude of cellular processes. Across multiple orders and families, many viruses have also been found to express small and long ncRNA species. Among these, microRNAs (miRNAs) have received the most focused attention. miRNAs are ~22-nucleotide (nt)-long linear RNAs that regulate translation, typically by binding to mRNA 3′-untranslated regions (UTRs) through near-perfect miRNA seed sequence (nt 2 to 7) complementarity. In addition to miRNAs, researchers continue to uncover novel classes of ncRNAs, such as long noncoding RNAs (lncRNAs), vault RNAs, and extracellular RNAs. Indeed, some of the most abundantly transcribed gene products from viruses are unique ncRNAs. For example, the Epstein-Barr virus-encoded small RNAs 1 and 2 (EBER-1 and -2) are RNA polymerase III (Pol III)-transcribed 167- and 172-nt nuclear RNAs that are highly expressed in latently infected and tumor cells, exhibit significant secondary hairpin structures, and affect multiple cellular processes, including apoptosis, innate immune function, and transcription (1). Likewise, the adenovirus VAI and VAII RNAs are highly expressed, multifunctional ~160-nt Pol III transcripts that regulate important cellular processes, including protein kinase R (PKR) activation and translation (1). Although the *in vivo* functions of most viral ncRNA transcripts remain poorly understood, numerous *in vitro* studies support the concept that these are multifunctional elements that can impact a wide range of cellular processes.

Like EBV, MHV68 encodes several Pol III-transcribed ncRNAs (2, 3). Within the first 5.5 kb of the MHV68 genome lie eight tRNA-miRNA-encoded RNAs (TMERs). Each TMER is 200 to 250 nt long and harbors a predicted tRNA-like element and two downstream pre-miRNA hairpins (see Fig. S1 in the supplemental material). The TMER transcripts are processed by a noncanonical biogenesis pathway that utilizes tRNase Z and Dicer to generate mature miRNAs (4, 5). We and others have reported differential expression of full-length TMERs and their mature miRNAs *in vivo* (2, 4 – 7). In addition, we previously reported that viruses with all 8 TMERs mutated have a slight attenuation in latency and are highly attenuated for lethal pneumonia (6, 8). However, the specific *in vivo* functions of individual TMERs are unknown.

10.1128/mSphere.00105-15.3 Figure S1  Predicted secondary structure of TMER4, based on the *in silico* Mfold prediction. Download Figure S1, EPS file, 4.1 MB. Copyright © 2016 Feldman et al. 2016 Feldman et al. This content is distributed under the terms of the Creative Commons Attribution 4.0 International license.

Notably, while EBV and MHV68 exhibit highly restricted gene expression programs during latency, both the EBV EBERs and the MHV68 TMERs are highly expressed in both latently infected cells and virus-associated tumors (2, 9), leading to speculation that these molecules play essential roles in the *in vivo* biology of gammherpesviruses. To elucidate the functions of specific TMERs, we generated a panel of MHV68 recombinants lacking pre-miRNA stem-loops within individual TMER transcripts. Following comprehensive *in vivo* analyses, we identified a crucial miRNA-independent function of TMER4 in MHV68 hematogenous dissemination and latency establishment.

## RESULTS

### TMER4 is required for splenic latency and pathogenesis.

**FIG 1:**
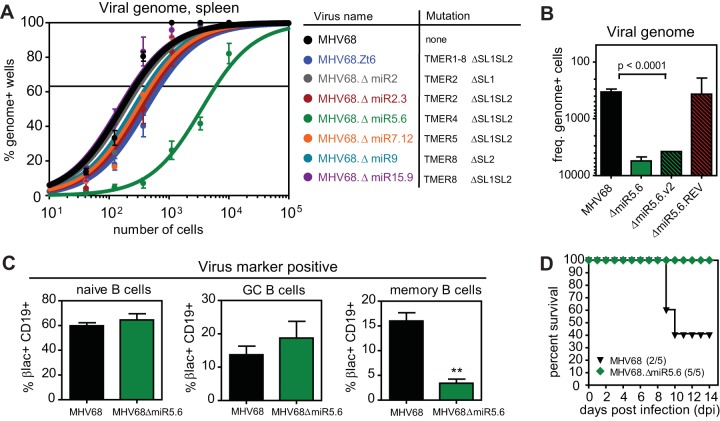
A TMER4 mutant that is highly attenuated for latency and pathogenesis. (A) Results of a limiting dilution nested PCR for the viral genome. Three B6 mice per group per experiment (3 experiments) were infected with 10^4^ PFU wild-type MHV68 (MHV68.βla) or MHV68 TMER stem-loop mutant. At 16 dpi, splenocytes were harvested, pooled within groups, and analyzed. The frequency of splenocytes harboring viral DNA was determined by Poisson distribution. Mutated pre-miRNAs (miR) and corresponding TMER stem-loops (SL) are indicated. (B) Reciprocal frequencies of viral genome-positive cells for similar experiments using additional viruses. (C) Subset distribution of MHV68-infected splenic B cells. Four B6 mice per group per experiment (3 experiments) were infected with wild-type (MHV68.βla; black) or TMER4 mutant virus (MHV68.ΔmiR5.6; green). At 16 dpi, splenocytes were harvested and stained with antibodies to CD19, IgM, and CD38, plus β-lactamase substrate CCF4/AM. Values are virus-positive CD19^+^ B cells expressing naive (IgM^+^), germinal center (IgM^−^ CD38^−/low^), or memory (IgM^−^ CD38^+^) markers. (D) Percent survival from virus-associated lethal pneumonia. BALB/c IFN-γ^−/−^ mice were infected with 4 × 10^5^ PFU virus and monitored for development of lethal respiratory disease.

To elucidate the function of individual TMERs in MHV68 latency and pathogenesis, we generated a panel of recombinant MHV68 viruses with precise single or double pre-miRNA stem-loop mutations of a single TMER transcript (Fig. 1A; see also Table S1 in the supplemental material). To assess the effects of individual TMER mutations on latency, we infected wild-type C57BL6/J (B6) mice and determined the frequency of latently infected splenocytes. B6 mice were inoculated intranasally (i.n.) with wild-type MHV68 (MHV68.βla marker virus), control MHV68 mutated in all 8 TMERs (MHV68.Zt6), or viruses with mutations in TMER2, TMER4, TMER5, or TMER8. Following clearance of acute replication and establishment of latency at 16 days postinfection (dpi), splenocytes were subjected to a limiting dilution nested PCR (6, 10). Preformed virus and reactivation assays were performed on parallel samples to verify latent infection.

10.1128/mSphere.00105-15.1 Table S1  MHV68 TMER recombinant viruses generated by two-step Red-mediated lambda recombination Download Table S1, PDF file, 0.1 MB. Copyright © 2016 Feldman et al. 2016 Feldman et al. This content is distributed under the terms of the Creative Commons Attribution 4.0 International license.

Consistent with our previous findings (6, 11), wild-type virus established latency in 1 in 250 splenocytes, while combinatorial TMER mutant MHV68.Zt6 displayed a subtle 2.8-fold attenuation (1 in 690). In concordance with these results, MHV68 with mutations of individual transcripts TMER2, TMER5, or TMER8 demonstrated frequencies that ranged between that of wild-type virus and the combinatorial mutant virus (Fig. 1A; see also Table S2 in the supplemental material). Unexpectedly though, the TMER4 mutant MHV68.ΔmiR5.6 demonstrated a severe 26-fold attenuation in latency establishment (1 in 6,500), despite carrying a lesion identical to the MHV68.Zt6 TMER4 mutation. The MHV68.ΔmiR5.6 phenotype was not due to altered expression of surrounding genes, as M1, M2, and M3 were all expressed at levels nearly equivalent to that of wild-type MHV68 (see Fig. S2 in the supplemental material). To experimentally validate these results, we generated a second virus carrying an identical TMER4 mutation and a revertant virus in which the wild-type TMER4 sequence was recombined back into MHV68.ΔmiR5.6. Importantly, the independently generated TMER4 mutant displayed a similar defect in latency (1 in 4,000), while the revertant virus established latency nearly equivalent (1 in 300) to wild-type virus (Fig. 1B), confirming the significance of TMER4 for latent infection.

10.1128/mSphere.00105-15.2 Table S2  Frequencies of genome-positive splenocytes for individual TMER mutant viruses (frequencies correspond to data presented in Fig. 1A) Download Table S2, PDF file, 0.1 MB. Copyright © 2016 Feldman et al. 2016 Feldman et al. This content is distributed under the terms of the Creative Commons Attribution 4.0 International license.

10.1128/mSphere.00105-15.4 Figure S2  MHV68.ΔmiR5.6 expresses normal levels of M1 and M2 and M3. qRT-PCR was performed on RNA isolated from NIH 3T12 fibroblasts at 18 h following infection with wild-type MHV68 (MHV68.βla) or MHV68.ΔmiR5.6 at an MOI of 5. Expression is relative to results with glyceradehyde-3-phosphate dehydrogenase (GAPDH) controls (×10^3^). Values represent means of 3 experiments ± standard deviations. Download Figure S2, EPS file, 1.4 MB. Copyright © 2016 Feldman et al. 2016 Feldman et al. This content is distributed under the terms of the Creative Commons Attribution 4.0 International license.

Like EBV and KSHV, MHV68 establishes latent infection in multiple B cell subsets. Studies with both EBV and MHV68 have led to the hypothesis that these viruses initially infect naive B cells and drive their differentiation into memory B cells independent of antigen. To determine whether TMER4 mutation disproportionately infected any specific B cell subsets, we employed flow cytometric analysis using a combination of staining for B cell markers and a fluorescent β-lactamase substrate. β-Lactamase is expressed by the parental virus and by all mutant viruses as a C-terminal fusion to episomal maintenance protein mLANA, facilitating facile identification of latently infected cells (11). Wild-type MHV68 was detected in all three B cell subsets, with a distribution of 59.8% naive, 13.7% germinal center, and 16.0% memory B cells (Fig. 1C). Notably, while MHV68.ΔmiR5.6 was detected in an increased percentage of naive (64.5%) and germinal center (18.7%) B cells, memory B cell infection was significantly reduced (3.2%), suggesting that TMER4 may play an essential role in MHV68-mediated B cell maturation.

Gammaherpesvirus infection of immunodeficient hosts is associated with a wide variety of pathogenic outcomes, including but not limited to lymphoproliferative diseases, lymphoma, vasculitis, and nonbacterial pneumonia. To determine whether TMER4 contributes to gammaherpesvirus pathogenesis, we infected gamma interferon (IFN-γ)-deficient mice, which develop lethal pneumonia following MHV68 infection (12). In contrast to wild-type MHV68 infection, which resulted in survival of only 40% of infected mice (Fig. 1D), MHV68.ΔmiR5.6 mice were completely attenuated for lethal pneumonia, with survival of 100% of infected animals. Thus, together these data demonstrated that TMER4 is an essential determinant of wild-type MHV68 latency and viral pathogenesis.

### TMER4 miRNAs are dispensable for latency.

**FIG 2:**
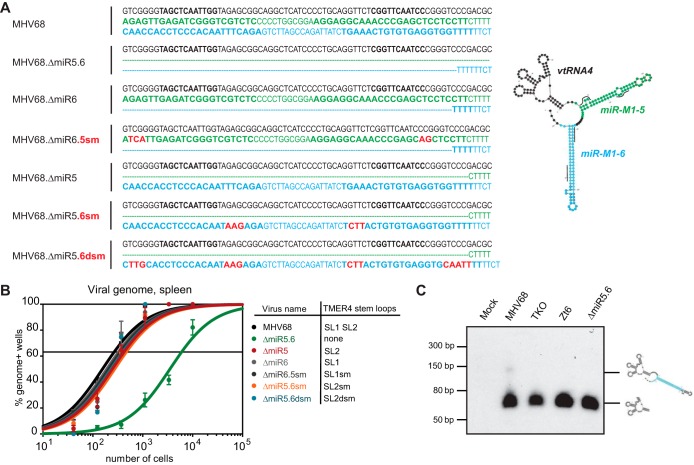
TMER4 miRNAs are not required for splenic latency. (A) Sequences of TMER4 mutant viruses. Nucleotides on the first line (black) correspond to vtRNA4, those on the second line (green) correspond to miR-M1-5 pre-miRNA, and those on the third line (blue) correspond to miR-M1-6 pre-miRNA. Red indicates locations of miRNA see sequence mutations. Lines on the TMER4 mFold predicted structure indicate miRNA seed sequence locations. (B) A limiting dilution nested PCR for viral genome was performed on B6 splenocytes exactly as described for Fig. 1. (C) Northern blotting for TMER4 vtRNA-linked transcripts was performed on RNA isolated from NIH 3T12 fibroblasts infected for 18 h. Probe was complementary to vtRNA4. *In silico*-predicted structures of vtRNA4 and vtRNA4 plus one stem-loop are indicated.

To determine whether the critical TMER4 function was conveyed by the TMER4-encoded miRNAs *mghv-M1-miR-5-5p* or *-6-3p*, we constructed a panel of additional TMER4 single stem-loop and miRNA seed sequence mutants (Fig. 2A). All mutations were designed *in silico* to maintain correct TMER4 tRNA (vtRNA4) and single stem-loop folding (see Fig. S3 in the supplemental material), and mutants were validated for expression of remaining miRNAs (see Fig. S4 in the supplemental material). Surprisingly, viruses encoding vtRNA4 plus either single stem-loop demonstrated normal latency (Fig. 2B). Further mutation of miRNA seed sequences within either remaining stem-loop did not compromise TMER4 function, unequivocally demonstrating that TMER4 miRNAs are dispensable for latency. In conjunction with the attenuated phenotype of the double stem-loop mutant MHV68.ΔmiR5.6, these findings strongly suggest that wild-type TMER4 functionality is conveyed by vtRNA4 plus a single stem-loop of nonspecific sequence. Consistent with this conclusion, northern blotting for vtRNA4 did not efficiently detect full-length TMER4, but instead revealed the presence of an approximately 150-nt stable TMER4 intermediate (Fig. 2C).

10.1128/mSphere.00105-15.5 Figure S3  Diagrams of *mghv-M1-miR-5* and -*6* stem-loops based on *in silico* predictions. The locations of miRNA seed sequences (lines and green dots) and seed mutations (red dots) are indicated. Download Figure S3, EPS file, 2.1 MB. Copyright © 2016 Feldman et al. 2016 Feldman et al. This content is distributed under the terms of the Creative Commons Attribution 4.0 International license.

10.1128/mSphere.00105-15.6 Figure S4  TMER4 miRNAs are expressed normally from TMER4 mutant viruses. TaqMan qRT-PCR was performed on RNA isolated from NIH 3T12 fibroblasts at 18 h following infection with wild-type MHV68 or mutant viruses (multiplicity of infection, 5). RT of mature miRNAs was performed using miRNA-specific stem-loop RT primers. Expression is relative to sno234 controls (×10^3^). Values represent means of 3 experiments ± standard deviations. Download Figure S4, EPS file, 1.4 MB. Copyright © 2016 Feldman et al. 2016 Feldman et al. This content is distributed under the terms of the Creative Commons Attribution 4.0 International license.

### TMER4 is required for hematogenous dissemination.

**FIG 3:**
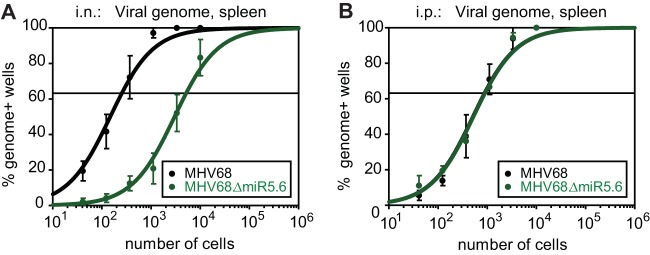
Latency attenuation of the TMER4 mutant is dependent upon the route of inoculation. Parallel groups of mice were infected i.n. (A) or i.p. (B) with wild-type MHV68 (MHV68.βla) or MHV68 TMER4 mutant (MHV68.ΔmiR5.6), and a limiting dilution nested PCR for viral genome was performed on splenocytes exactly as described for Fig. 1.

To test the hypothesis that TMER4 facilitated latent infection of susceptible cells at peripheral sites such as the spleen, we compared splenic latency following i.n. inoculation to that obtained following intraperitoneal (i.p.) inoculation, a route which bypasses typical mucosal immune responses and dissemination requirements. Interestingly, while the TMER4 mutant MHV68.ΔmiR5.6 exhibited a 26-fold latency defect following i.n. inoculation, when administered i.p. the same mutant established splenic latency at a level equivalent to wild-type virus (Fig. 3). Thus, these results demonstrated that TMER4 is not essential for latency establishment at peripheral sites, but instead suggested that this ncRNA may perform a critical function at sites of acute replication or at mucosal immune barriers.

**FIG 4:**
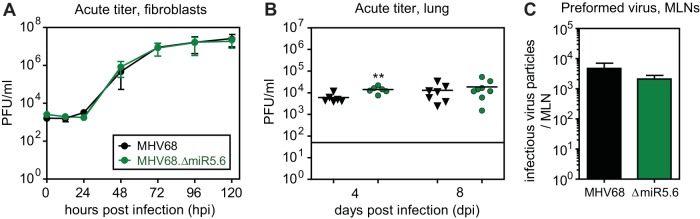
TMER4 mutant virus undergoes normal acute replication in lungs and mediastinal lymph nodes. (A) Lytic replication in fibroblasts *in vitro*. Growth curves were generated after infection with NIH 3T12 fibroblasts at an multiplicity of infeciton of 0.05, and titers were determined by plaque assay. (B) Titers of virus in the lungs of B6 mice were determined by plaque assay at 4 and 8 dpi. (C) Number of preformed infectious virus particles in the MLN of B6 mice at 8 dpi was determined by a limiting dilution preformed virus assay.

To determine whether TMER4 contributed to the acute phase of infection, we quantified lytic replication *in vitro* and *in vivo*. In multistep growth curve experiments for fibroblasts, MHV68.ΔmiR5.6 replicated identically to wild-type virus (Fig. 4A). Likewise, following i.n. inoculation of B6 mice, the TMER4 mutant displayed no defect in acute replication in the lungs (Fig. 4B), with titers equivalent to or slightly higher than those with wild-type virus. Subsequent to replication in the lungs, MHV68 spreads to lung-draining mediastinal lymph nodes (MLN), likely via dendritic cell trafficking (13, 14). To determine whether TMER4 was essential for trafficking to or replication in MLN, we quantified preformed virus, as this assay is up to 50 times more sensitive than plaque assays (10). Consistent with normal lung replication, MHV68.ΔmiR5.6 infectious particles were present in MLN at 8 dpi at a level nearly equivalent to that of wild-type virus (Fig. 4C). Thus, these findings clearly demonstrated that TMER4 is not required for acute replication in, or lymphatic spread to, the lung-draining lymph nodes.

**FIG 5:**
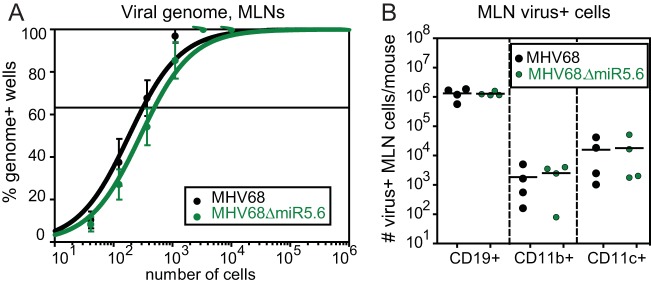
TMER4 mutant virus establishes normal latency in the mediastinal lymph node. (A) A limiting dilution nested PCR for viral genome was performed on B6 MLN cells at 16 dpi, exactly as described for Fig. 1. (B) The absolute number of virus-positive MLN cells at 16 dpi was determined by antibody surface marker and CCF4/AM β-lactamase substrate staining.

Subsequent to myeloid cell trafficking to the MLN, MHV68 establishes latency in MLN B cells, macrophages, and dendritic cells. To determine whether MHV68.ΔmiR5.6 undertook normal latent infection at this site, we quantified latency by limiting dilution PCR and quantified infected MLN cell types by multiparametric flow cytometry. In contrast to peripheral sites, the TMER4 mutant virus established latency in the MLN at a frequency (1 in 500) nearly equivalent to that of the wild-type virus (1 in 300) (Fig. 5A). Consistent with this, the number of virus-infected CD19^+^, CD19^−^CD11b^+^CD11c^−^, and CD19^−^CD11b^−^CD11c^+^ cells did not differ significantly between MHV68.ΔmiR5.6 and wild-type MHV68 (Fig. 5B). Together, these results demonstrated that TMER4 is not required for latency establishment at lymphoid sites proximal to sites of acute replication.

**FIG 6:**
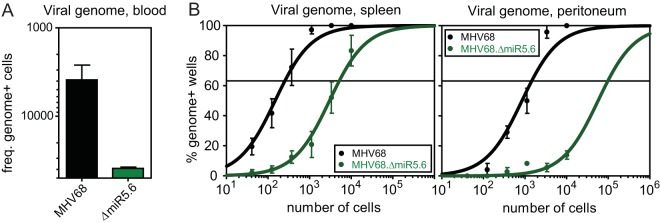
TMER4 mutant virus is highly attenuated for hematogenous dissemination to sites of peripheral latency. (A) Blood leukocytes from B6 mice at 6 dpi were subjected to a limiting dilution nested PCR for the viral genome, exactly as described for Fig. 1. Results indicate the reciprocal frequencies of viral genome-positive cells. (B) At 16 dpi, a limiting dilution nested PCR for viral genome was performed on B6 peritoneal cells and splenocytes, exactly as described for Fig. 1.

Following spread to the MLN, MHV68 disseminates to peripheral sites likely through hematogenous circulation of infected B cells (15 – 17). To determine whether TMER4 was essential for MHV68 peripheral dissemination, we quantified the frequency of blood leukocytes carrying viral genome at 6 dpi. At this time point, virus titers at peripheral sites remain below the level of detection, yet virus particles can be detected in splenic marginal zone macrophages, indicative of the earliest stages of peripheral virus seeding (17). In stark contrast to the equivalent replication and latency observed in the local MLN, TMER4 mutant virus genome was detected in only 1 in 40,000 circulating leukocytes, compared to 1 in 2,600 for wild-type virus (Fig. 6A), suggesting that the mutant virus did not efficiently mobilize to the vasculature. In accordance with this conclusion, MHV68.ΔmiR5.6 was similarly attenuated for latency in both the spleen and peritoneum, two well-characterized sites of latent MHV68 infection (Fig. 6B). Thus, cumulatively these results demonstrated that TMER4 is required for hematogenous dissemination to peripheral sites of latency.

## DISCUSSION

Viral ncRNAs are expressed by a diverse array of viruses and are thought to regulate a multitude of host and viral processes. Further, the evolution of high-throughput sequencing and bioinformatic algorithms has led to the identification of hundreds of new putative viral ncRNAs. However, the *in vivo* relevance of most of these transcripts remains unknown. Like the EBV EBERs, the MHV68 TMERs are abundantly expressed *in vivo* in latently infected cells and hyperplastic lesions, leading to speculation that these ncRNAs have critical functions in latency and oncogenesis. Through the use of viral mutagenesis and comprehensive *in vivo* testing, we have now identified one of these ncRNAs, TMER4, as an essential determinant of hematogenous dissemination and peripheral latency establishment of wild-type MHV68.

### Role of TMER4 in dissemination.

**FIG 7:**
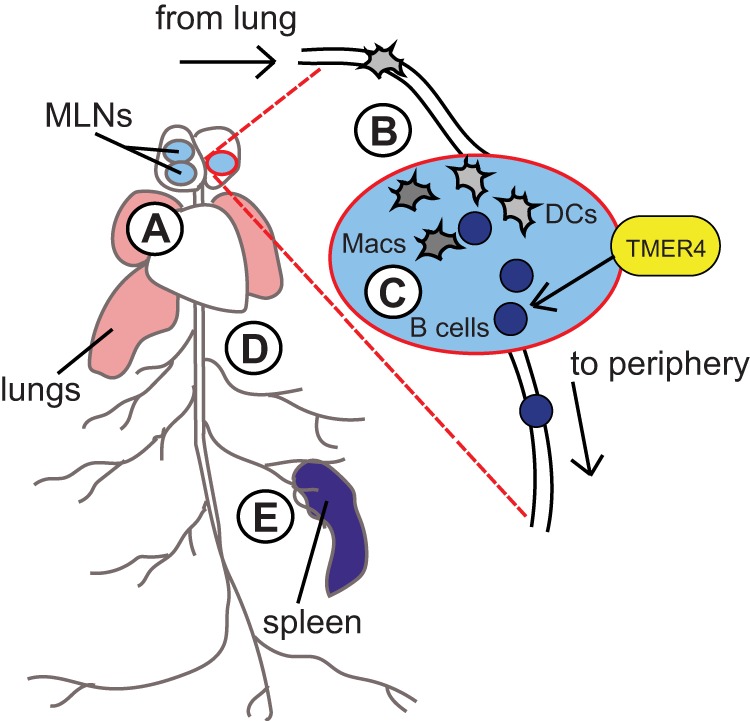
Working model for TMER4 ncRNA’s essential role at a key MLN bottleneck to facilitate hematogenous dissemination of MHV68. The TMER4 double stem-loop mutant displays normal acute replication in the lungs (A), normal trafficking to and acute replication in the MLN (B), and normal leukocyte distribution and latency establishment in the MLN (C). However, the TMER4 mutant is highly attenuated for infection of circulating leukocytes (D) and highly attenuated for latent infection at peripheral sites (E). Therefore, we propose that the TMER4 ncRNA mediates an essential function at a critical viral dissemination bottleneck in the MLN. Because B cells are required for MHV68 trafficking to peripheral sites, the key dissemination bottleneck is likely related to (i) intrinsic or (ii) extrinsic stimulation of B cell egress from MLN or (iii) promotion of survival of infected MLN B cells.

Following inoculation, MHV68 replicates in local tissues and then spreads systemically to establish latency at peripheral sites. Although the specific molecular details by which these events occur are not well understood, it appears that MHV68 utilizes typical routes of pathogen dissemination (Fig. 7). The virus initially undergoes acute lytic replication in the lung epithelial cells, typically peaking around 7 to 8 days. During this time, the virus transits to lung-draining MLN, likely via dendritic cell transport. Following requisite passage through dendritic cells (13, 14), the virus infects other leukocytes in the MLN, including B cells. It is well-established that B cells are required for systemic spread of the virus (15 – 17), suggesting that infected B cell egress from the MLN is a key dissemination checkpoint. Consistent with this possibility, free virus is typically not detectable in the circulation. Further, latency establishment in the spleen is mediated by marginal zone B cell uptake of free virus and subsequent transfer to follicular B cells (17), strongly suggesting that infection at peripheral sites is dependent upon virus reactivation from circulating cells.

Based on our observation that the TMER4 mutant MHV68.ΔmiR5.6 underwent normal lytic replication in the lungs and the lung-draining lymph nodes, we concluded that TMER4 is dispensable for both acute replication and MLN trafficking. In accordance with this, we found that MHV68.ΔmiR5.6 established near-normal frequencies of latent infection in the MLN. In contrast though, the frequencies of infection in circulating leukocytes and at downstream peripheral sites were significantly diminished (15- to 50-fold), strongly suggesting that egress from the MLN is the critical bottleneck for MHV68.ΔmiR5.6 dissemination (Fig. 7). Thus, our findings implicate TMER4 as a key mediator of infected cell survival or egress from the local draining lymph node.

### TMER4 function.

In the MLN, it is possible that TMER4 acts directly as an effector molecule or more indirectly as an antagonist or suppressor of other TMERs. A surprising finding was revealed by direct comparison of the TMER4 mutant MHV68.ΔmiR5.6 to the combinatorial mutant MHV68.Zt6, which carries an identical TMER4 mutation in addition to mutations in the seven remaining TMERs. While the TMER4 mutant exhibited highly attenuated latency establishment *in vivo*, the combinatorial TMER mutant displayed only a subtle latency defect. These results likely indicate that TMER4 counteracts deleterious consequences of one or more of the other TMERs. For instance, it is possible that TMER4 turns off virus-repressive pathways that are activated by other TMERs or TMER-encoded miRNAs. A classic example of such a scenario is the highly pathogenic human papillomavirus, which uses E7 to drive cell cycle progression and E6 to block the deleterious consequence of p53-mediated apoptosis.

As a direct effector, TMER4 may (i) provide a vital prosurvival signal or block apoptosis of MHV68-infected cells, (ii) suppress a key antiviral immune response that blocks systemic infection, or (iii) initiate an immune response necessary for egress of MHV68-infected cells into the peripheral circulation. It is noteworthy that the EBV EBERs and adenovirus VA RNAs, Pol III-transcribed viral RNAs of approximately the same size as the partially processed TMER4, each convey some of these functions. For example, the EBERs block IFN-mediated apoptosis of infected cells, and VA RNAI blocks IFN-induced activation of PKR (1). Although it remains possible that TMER4 suppresses T cell function, antiviral CD8 T cell responses were normal during MHV68.ΔmiR5.6 infection (data not shown), suggesting that adaptive immune function was largely intact. In preliminary experiments, we have not detected direct TMER4 activation of innate immune sensors (data not shown); however, this does not rule out other potential mechanisms of TMER-mediated innate immune induction. Consistent with this possibility, recent findings implicate host lncRNA functions in innate and adaptive immunity (18); thus, it is plausible that TMER4-induced alterations in the intrinsic or extrinsic immune environment could lead to upregulation of B cell egress markers, such as the sphingosine-1-phosphate (S1P) receptor. Unfortunately, due to the low number of infected cells in circulation relative to the number of infected cells in the MLN, detection of accumulated MHV68.ΔmiR5.6-infected cells in the MLN is impossible.

It is also plausible that TMER4 functions to directly antagonize or suppress other TMERs. For example, TMER4 may compete for binding of other TMERs to a sensor molecule that activates downstream pathways which prevent virus dissemination. Although it is also conceivable that TMER4 directly suppresses expression of other TMERs, we have not noted any reduction in expression or processing of other TMERs in the absence of TMER4.

Owing to the complexity of pneumonia pathogenesis, the specific contribution of TMER4 to induction of this disease is unclear; however, it is likely that the role of TMER4 in pneumonia pathogenesis is distinct from its function in dissemination. We previously demonstrated that mutants deficient in a combination of TMERs are highly attenuated for pneumonia lethality (6, 8), arguing that TMER4 does not counteract the effect of other TMERs in this model. Confounding such an interpretation, viruses which express TMER1 or vtRNA1 but do not express other TMERs display partial restoration of disease (8), strongly suggesting that vtRNA1 is a key contributor to pathogenesis. Further, pneumonia induction by MHV68 likely reflects a combination of contributions from acute virus replication, latent virus reactivation, and immune induction (12). Thus, the specific function of TMER4 in pneumonia pathogenesis remains unclear.

### Putative TMER4 structure and role as a polyfunctional ncRNA.

The mutant virus used in these studies, MHV68.ΔmiR5.6, lacks both of the TMER4 pre-miRNA stem-loops but retains the full Pol III promoter, stop, vtRNA, and intervening sequences. The observation that MHV68.ΔmiR5.6 expresses levels of the TMER4 vtRNA equivalent to wild-type virus suggests that vtRNA4 alone is not sufficient to convey wild-type function. Surprisingly though, restoration of either stem-loop resulted in normal latency establishment, and further mutation of miRNA seed sequences revealed no obvious constraint to the stem-loop nucleotide sequence. Thus, these results revealed that TMER4 functions as a unique ncRNA element, likely comprised of vtRNA4 plus one stem-loop of nonspecific sequence. Notably, we did not detect robust expression of the 203-nt full-length TMER4, but instead we detected a TMER4 intermediate approximately 150 nt long, consistent with the size of vtRNA4 plus a single stem-loop (~145 nt).

Based on these collective results, we hypothesize that vtRNA4 dictates specificity of TMER4 function. It is likely that the requirement for additional stem-loop sequence may be structural, as has been observed for several host lncRNAs, which have notoriously poor sequence conservation but display evolutionary conservation of structure and function (19). Thus, the TMER4 stem-loop may act as a structural element to facilitate protein binding, RNA processing, or localization. For example, all TMER4 transcripts retaining a stem-loop would be expected to interact with components of the RNA-induced silencing complex (RISC) machinery, independent of sequence specificity.

Although the TMER4 miRNAs were dispensable for latency establishment, our results do not exclude the possibility that the MHV68 miRNAs have a functional role during infection. We and others have previously demonstrated the expression of mature *mghv-M1-miR-5-5p* and *mghv-M1-miR-6-3p* miRNAs during infection (4, 6, 7), indicating miRNA processing of at least some TMER4 transcripts. Thus, it is conceivable that some or all of the TMER transcripts are polyfunctional ncRNAs, as has already been described for TMER1 (8). It is also formally possible that TMER4 encodes a small peptide; however, the lack of sequence specificity required for the TMER4 stem-loop makes this scenario unlikely.

In the work described here, we have defined an MHV68 ncRNA that plays an essential role in virus dissemination and subsequent peripheral latency establishment. We have determined that TMER4 plays a crucial role in the local draining lymph node, most likely through promoting the survival or egress of infected B cells that are requisite for hematogenous dissemination of the virus. Future work will be necessary to determine the specific molecular mechanism by which TMER4 functions and whether similar strategies have been adopted by other viruses. However, amid the myriad of putative ncRNAs recently discovered, the findings presented here provide insight into the type of potent pathogenic activity that can be conveyed by a single viral ncRNA *in vivo*.

## MATERIALS AND METHODS

### Cell lines.

NIH 3T12 murine fibroblasts (ATCC CCL-164) and murine embryonic fibroblasts (MEFs) from C57BL/6J mice (GSC-6002; GlobalStem) were maintained in Dulbecco’s modified Eagle’s medium (DMEM; 10013CM; Corning) supplemented with 10% fetal calf serum, 100 U/ml penicillin, and 100 mg/ml streptomycin.

### Viruses.

Parental bacterial artificial chromosome (BAC)-derived wild-type MHV68 (20) and MHV68.ORF73βla (11) have been previously described. Mutant viruses were generated in three stages using two-step Red-mediated recombination (21) onto a wild-type MHV68.ORF73βla BAC backbone. Briefly, PAGE-purified primers with sequence homologous to both the viral sequence flanking the desired mutation site and the kanamycin (Kan) resistance gene were used to amplify the Kan gene. The forward primer, including the Kan sequence, also contained an I-SceI homing endonuclease cutting site. The resulting amplicon was purified and electroporated into GST1783 *Escherichia coli* cells containing the MHV68.ORF73βla BAC backbone and Red recombinase machinery. Transformed cells were recovered and grown at 30°C overnight on Kan selection medium. DNA extracted from the resulting colonies was isolated and digested with XhoI and then analyzed by pulse-field gel electrophoresis (PFGE) to screen for primer insertion and to confirm genomic integrity. Positive clones were subjected to I-SceI-mediated recombination, which was induced by 1% arabinose. Mutants were validated by PFGE, PCR, and sequencing. BAC DNA from a single positive clone was then isolated and transfected into NIH 3T12 cells by using a TransIT-3T3 transfection kit (MirusBio). Resulting virus was passaged twice and amplified on Cre recombinase-expressing NIH 3T12 cells to remove the BAC cassette. Titers of final viral BAC-minus stocks were determined by plaque assay on NIH 3T12 cells.

### Mice, infections, and cell harvests.

Female C57BL/6J (B6) mice were purchased from Jackson Laboratory (Bar Harbor, ME) at 7 to 8 weeks of age and housed in a biosafety level 2+ (BSL2+) facility at the University of Florida (Gainesville, FL) in accordance with all federal guidelines and as approved by the University of Florida Institutional Animal Care and Use Committee. For i.n. inoculations, B6 mice (Jackson Laboratory) were anesthetized with isoflurane and then inoculated with 10^4^ PFU of virus in 30 µl serum-free DMEM. BALB.IFN-γ^−/−^ mice (Jackson Laboratories) were inoculated with 4 × 10^5^ PFU i.n. For i.p. inoculations, B6 mice were injected with 10^3^ PFU virus in 500 µl serum-free DMEM. Unless otherwise noted, groups of three mice were used per time point, per experiment, for all studies. For blood harvests, blood was collected by heart puncture and placed into BD Microtainers with lithium heparin (catalog number 365965; BD). Buffy coats were separated using lympholyte-M cell separation medium (cI5030; Cedarlane).

### Latency assays.

Limiting dilution nested PCR was performed on 3-fold serial dilutions of total cells from spleen, peritoneum, mediastinal lymph nodes, and blood (10). Briefly, following isolation, cells were washed, counted, and resuspended in an isotonic buffer. Cells were serially diluted 3-fold in a background of uninfected RAW 264.7 murine macrophages, such that a total of 10^4^ cells were present in each PCR mixture. Cells were plated in a 96-well plate at 12 wells per dilution, and dilution series of positive controls for single-copy sensitivity and negative controls were plated in parallel. Following plating, cells were lysed with proteinase K, nested PCR was performed using primers specific for MHV68 ORF72, and amplicons were visualized on a 3% agarose gel. Limiting dilution assays were also used to determine the frequency of reactivation from latency or preformed virus by plating 2-fold serial dilutions of intact or mechanically disrupted cells over a monolayer of mouse embryonic fibroblasts (10). Infected cell subsets were identified and characterized by flow cytometry using antibodies and CCF4-AM β-lactamase substrate, as previously described (11). Briefly, following cell isolation, red blood cells were lysed, then samples were blocked with anti-mouse CD16/CD32 (Fc block; BD Biosciences). Cells were stained with rat anti-mouse CD19-allophycocyanin (APC)/Cy7 (clone ID3; BD Biosciences), AA4-APC (clone AA4.1; eBiosciences), IgM-APC (clone II/41; BD Biosciences), and/or CD38-Alexa Fluor 700 (clone 90; eBiosciences). Subsequently, cells were washed and resuspended in freshly prepared CCF4-AM β-lactamase substrate (LiveBLAzer FRET B/G reagent; Life Technologies). Following washing, cells were analyzed on a BD LSR II flow cytometer using the following schemes: naive follicular B cells (CD19^+^ IgM^+^), germinal center B cells (CD19^+^ IgM^−^ CD38^low/−^), and memory B cells (CD19^+^ IgM^−^ CD38^+^); or B cells (CD19^+^), macrophages (CD19^−^CD11b^+^CD11c^−^), and dendritic cells (CD19^−^CD11b^−^CD11c^+^). Unstained, fluorescence-minus-one, and isotype-stained controls were included in each experiment.

### RNA assays.

As previously described (11), for all of the TaqMan stem-loop quantitative reverse transcription-PCR (qRT-PCR) assays, a total of 2 × 10^6^ infected NIH 3T12 fibroblasts were lysed using the TaqMan microRNA Cells-to-CTTM kit (catalog number 4391848; Life Technologies). Next, 5 µl of sample RNA was added to the RT reaction mixture, which was performed using custom stem-loop primers provided with the Custom TaqMan small RNA assay. Stem-loop primers were designed using mature MHV68 miRNA sequences as detailed on miRBase 19.0 with its supplement of previously published mature miRNA reads. PCR was performed using 1.33 µl of the RT reaction mixture with 17.67 µl of the TaqMan PCR master mix (no UNG) and 1 µl of corresponding mature miRNA TaqMan probe, according to the manufacturer’s instructions. For Northern blotting, cell samples were collected in phenol, isoamyl alcohol, guanidinium isothiocyanate, and beta-mercaptoethanol (PIG-B) solution, pH 4.5. Subsequently, RNA in lysis solution was mixed with chloroform, and the aqueous phase was collected and then precipitated with isopropanol. Following a wash with ice-cold 75% ethanol, total RNA was resuspended in diethyl pyrocarbonate deionized water and stored at −80°C until use. RNA (10 µg) was loaded onto a urea denaturing 15% acrylamide gel, and then the samples were transferred to a HybondN^+^ membrane. Following cross-linking, blots were hybridized to purified [γ-^32^P]ATP-labeled 25-nt DNA probes, washed, and then exposed to film.

### Plaque assays.

Plaque assays were performed as previously described (6, 10). Briefly, harvested lungs were placed in sterile 2-ml screw-cap tubes containing 1 ml DMEM and 500 µl of 1-mm zirconia-silica beads (BioSpec Products) and stored at −80°C until use.

Samples were thawed on ice and then homogenized with a mini-beadbeater (BioSpec Products). Subsequently, samples were serially diluted 10-fold in serum-free DMEM, added to a single well of a 6-well plate containing a monolayer of NIH 3T12 fibroblasts, and then overlaid with a 1:1 mixture of methylcellulose (Sigma) and 2× Temin’s minimal essential medium modification, without phenol red (Invitrogen) and supplemented with 10% fetal calf serum, 100 U/ml penicillin, 100 mg/ml streptomycin. After 7 days, neutral red was used for plaque quantification.

### Statistical analysis.

Percentages of β-lactamase-positive B cell subsets were statistically analyzed using a one-way unpaired Student’s *t* test. Frequencies of viral genome-positive cells were determined from nonlinear regression analysis of sigmoidal dose-response best-fit curve data. Based on a Poisson distribution, the frequency at which at least one event in a given population is present occurs at the point where the regression analysis line intersects 63.2%. All data were analyzed using Prism software (GraphPad Software, San Diego, CA).
