# Supplementary material for: A Gammaherpesvirus Noncoding RNA Is Essential for Hematogenous Dissemination and Establishment of Peripheral Latency
Source: mSphere. 2016 Mar 2;1(2):e00105-15. doi: 10.1128/mSphere.00105-15 (PMC4838037; doi:10.1128/mSphere.00105-15)
Supplement: Table S1 [file sph002162019st1.pdf]

Virus Name	TMER	Mutation	Virus Backbone
MHV68.ΔmiR2.3	2	ΔSL1 ΔSL2	MHV68.ORF73βla BAC
MHV68.ΔmiR2	2	ΔSL1	MHV68.ORF73βla BAC
MHV68.ΔmiR5.6	4	ΔSL1 ΔSL2	MHV68.ORF73βla BAC
MHV68.ΔmiR5	4	ΔSL1	MHV68.ORF73βla BAC
MHV68.ΔmiR6	4	ΔSL2	MHV68.ORF73βla BAC
MHV68.ΔmiR5.6REV	4	+SL1+SL2	MHV68.ΔmiR5.6 BAC
MHV68.ΔmiR6.5sm	4	ΔSL2.miR*	MHV68.ΔmiR6 BAC
MHV68.ΔmiR5.6sm	4	ΔSL1.miR*	MHV68.ΔmiR5 BAC
MHV68.ΔmiR5.6dsm	4	ΔSL1.miR**	MHV68.ΔmiR5.6sm BAC
MHV68.ΔmiR7.12	5	ΔSL1 ΔSL2	MHV68.ORF73βla BAC
MHV68.ΔmiR15.9	8	ΔSL1 ΔSL2	MHV68.ORF73βla BAC
MHV68.ΔmiR9	8	ΔSL2	MHV68.ORF73βla BAC

Table S1. MHV68 TMER-recombinant viruses generated by two-step red-mediated lambda recombination.
