# Supplementary material for: A Gammaherpesvirus Noncoding RNA Is Essential for Hematogenous Dissemination and Establishment of Peripheral Latency
Source: mSphere. 2016 Mar 2;1(2):e00105-15. doi: 10.1128/mSphere.00105-15 (PMC4838037; doi:10.1128/mSphere.00105-15)
Supplement: Table S2 [file sph002162019st2.pdf]

Virus	TMER mutated	Frequency of genome+ cells
MHV68	None	1 in 250
MHV68.Zt6	1-8	1 in 690
MHV68.ΔmiR2<br>MHV68.ΔmiR2.3	2	1 in 320<br>1 in 575
MHV68.ΔmiR5.6	4	1 in 6500
MHV68.Δ7.11	5	1 in 500
MHV68.ΔmiR15.9<br>MHV68.ΔmiR9	8	1 in 250<br>1 in 430

Table S2. Frequencies of genome+ splenocytes for individual TMER mutant viruses. Frequencies correspond to data presented in Fig. 1A.
